# Comprehensive honey authentication in Bangladesh: Profiling physicochemical and bioactive compounds to distinguish floral sources and detect adulteration

**DOI:** 10.1016/j.heliyon.2024.e40203

**Published:** 2024-11-06

**Authors:** Roksana Al Nafiu Insha, Md Nahidul Islam, Joydeb Gomasta, Mohammad Nazmol Hasan, Md Ruhul Amin, Noor Shaila Sarmin, Md Mamunur Rahman

**Affiliations:** a Department of Entomology, Bangabandhu Sheikh Mujibur Rahman Agricultural University, Bangladesh; b Department of Agro-Processing, Bangabandhu Sheikh Mujibur Rahman Agricultural University, Bangladesh; c Institute of Food Safety and Processing, Bangabandhu Sheikh Mujibur Rahman Agricultural University, Bangladesh; d Department of Horticulture, Bangabandhu Sheikh Mujibur Rahman Agricultural University, Bangladesh; e Department of Statistics, Bangabandhu Sheikh Mujibur Rahman Agricultural University, Bangladesh; f Department of Agroforestry and Environment, Bangabandhu Sheikh Mujibur Rahman Agricultural University, Bangladesh

**Keywords:** Honey adulteration, Biochemical analysis, PCA, Correlation, Profiling

## Abstract

This study aims to advance honey authentication in Bangladesh by developing a reliable, cost-effective, and user-friendly system capable of distinguishing floral sources and purity. We analyzed various physicochemical parameters and bioactive compounds in honey samples from diverse floral sources across Bangladesh during different floral seasons, including deliberately adulterated samples. Results showed clear distinctions among the tested parameters. Notably, moisture content exhibited considerable variability, with the highest levels found in kholisha (24.07%–24.99 %) and the lowest in black cumin honey (17.80%–27.46 %). Jujube honey exhibited the highest protein content at 10 % adulteration, whereas mustard honey showed the least at 20 % adulteration. Generally, adulterated honey samples showed a considerable deviation in protein content compared to known standards for pure honey, suggesting adulteration significantly impacts protein levels. Black cumin honey showed the highest electrical conductivity, followed by jujube. Antioxidant levels were highest in litchi honey, followed by ayurvedic and kholisha. Multivariate analysis revealed that two principal components explained 65 % of total variances and could separate the various clusters of honey samples. The pH values, total phenolic, total flavonoid, protein content, electrical conductivity, color intensity at 360 nm and 635 nm, and antioxidant properties of all tested honey samples showed a strong positive correlation. Overall, the manuscript strategically combines experimental findings with detailed analyses to explore the complex nature of honey composition, adulteration issues, and the significance of authenticating honey to maintain quality and ensure consumer trust in the market. This study is the first to employ a comprehensive set of physicochemical and bioactive indicators with multivariate analysis for honey authentication in Bangladesh, offering a novel and systematic approach to identifying purity and floral sources.

## 1 Introduction

Honey is a widely consumed natural health promoting compound enjoyed globally for its rich nutritional content and various beneficial properties, including antioxidants, antimicrobial qualities, and prebiotics [1]. In addition to being consumed directly, honey is widely used in the food industry as a preservative and sweetener [2]. The production of honey significantly affects both society and biodiversity. An approximate 1.72 million tons of honey are produced globally, with China contributing the most, followed by Turkey and Canada [3]. Honey bees gather honey by gathering nectar from blossoms and secretions from living plant parts.

The composition of honey is significantly influenced by both its botanical and entomological origins, as it contains pollens and nectars from specific plants. Nectar honey and honeydew are the two primary forms of honey. Nectar is a sweet solution with a range of concentrations from 5 % to 80 %. Approximately 95 % of the solid components in honey consist of sugars, with the remaining portion comprising amino acids (0.05 %), minerals (ranging from 0.02 % to 0.45 %), small quantities of organic acids, vitamins, and aromatic compounds [[4], [5], [6]]. Natural honey consists of glucose, fructose, sucrose, water, minerals, amino acids, vitamins, pigments, and essential oils [7]. Hence, the botanical sources, whether mono-floral (from a single type of flower) or poly-floral (from multiple flower types), play a vital part in defining its physico-chemical and bioactive properties. Bioactive properties can vary due to differences in the botanical source and the types of honeybee species involved in its production. The European honeybee *Apis mellifera*, the Asian honeybee *Apis cerana*, and the giant honeybee *Apis dorsata* are the major honeybee in Bangladesh [8]. However, honey can be adulterated by mixing and falsely naming the botanical source [9]. Besides floral sources honey composition is governed by external factors such as season, environment, and processing methods [10].

Various factors, including moisture content, pH, electrical conductivity (EC), hydroxymethylfurfural (HMF) content, color, total phenolic content (TPC), flavonoid content (FC), antioxidants, flavanols, sugars, and organic acids, are essential determinants in authenticating honey and discerning both its entomological and botanical origins [11,12]. Unfortunately, adulterated and incorrectly labeled honey products are now a global problem [13]. A common method for producing adulterated honey is to mix sugar solution and various types of syrup in honey. Another unethical technique is to provide sugar and syrup to honeybees so they can create honey, which is then purposefully labeled with a particular flower or place of origin [14]. As a result, regulatory agencies, producers, retailers, and consumers are keen to determine the origin and quality of honey.

Despite the significant increase in honey consumption worldwide in recent years, the safety of honey is not consistently assessed and monitored. This has eroded consumer trust and interest in this valuable product, as adulteration now encompasses not only changing qualitative traits through mixing but also the mislabeling of mono-floral and poly-floral honey types and their associated bee species, as discussed by Schievano, Stocchero et al. [15]. Among the recognized *Apis* species, only two are commercially used: *A. mellifera* and *A. cerana*. In countries like Bangladesh, *A. cerana* honey has a higher price, typically three to five times that of *A. mellifera* honey, due to limited productivity and local consumer preferences.

The apiculture sector in Bangladesh is promising, therefore, authenticating the botanical origin of honey along with detecting adulteration are crucial to protecting consumer interests and fostering honey market development. Strict quality standards are required to protect consumer health and guarantee the authenticity of honey. Nonetheless, there is a worldwide disparity between the production and consumption of honey, which forces nations to boost production in order to meet both domestic and international demand for honey products. But due to the lack of proper authentication systems and appropriate labeling regarding floral sources and bee species, most honey produced in Bangladesh cannot be exported. This research aimed to provide a reference for authenticating pure and adulterated honeys based on their botanical sources through a comparative assessment of their physical and biochemical attributes.

While various honey authentication methods have been developed globally, including spectroscopic techniques, chromatography, DNA barcoding, and chemometric analyses, many of these methods are either costly, require sophisticated instruments, or are not user-friendly for widespread implementation by local producers and regulatory bodies. For instance, advanced spectroscopic techniques like Nuclear Magnetic Resonance (NMR) spectroscopy or Liquid Chromatography-Mass Spectrometry (LC-MS) are highly effective but often prohibitively expensive and technically demanding [16].

Despite extensive research on honey composition worldwide, the novelty of this study lies in its comprehensive approach to honey authentication using a diverse set of physicochemical and bioactive indicators in the context of Bangladeshi honeys. By integrating multivariate analysis techniques, this research not only differentiates between honey types based on floral sources but also systematically identifies adulteration, which has not been extensively done for honeys produced in Bangladesh. This study modernizes honey authentication in Bangladesh by providing a cost-effective, comprehensive, and user-friendly method that bridges significant gaps between global techniques and local implementation. This approach not only enhances the reliability of honey authentication but also paves the way for improving consumer trust and expanding the export potential of Bangladeshi honey.

The primary objective of this research is to establish a comprehensive honey authentication system in Bangladesh, which can reliably distinguish between different floral sources and detect purity levels. Therefore, this study aims to profile a series of physicochemical parameters and bioactive compounds across various honey samples from different floral sources in Bangladesh, deliberately adulterate honey samples to identify specific markers and methods for detecting adulteration, use multivariate analysis to determine the most significant parameters for distinguishing between pure and adulterated honey samples, and develop a user-friendly and cost-effective system that can be used by regulatory agencies, producers, and retailers to ensure the authenticity and quality of honey in the market.

## 2 Materials and methods

### 2.1 Sample collection, pretreatment and preservation

Honey samples were collected from various regions across Bangladesh, following a systematic approach to ensure representativeness. The collection sites were carefully selected to capture diverse floral sources and ensure a comprehensive sample set.

**Fig. 1:**
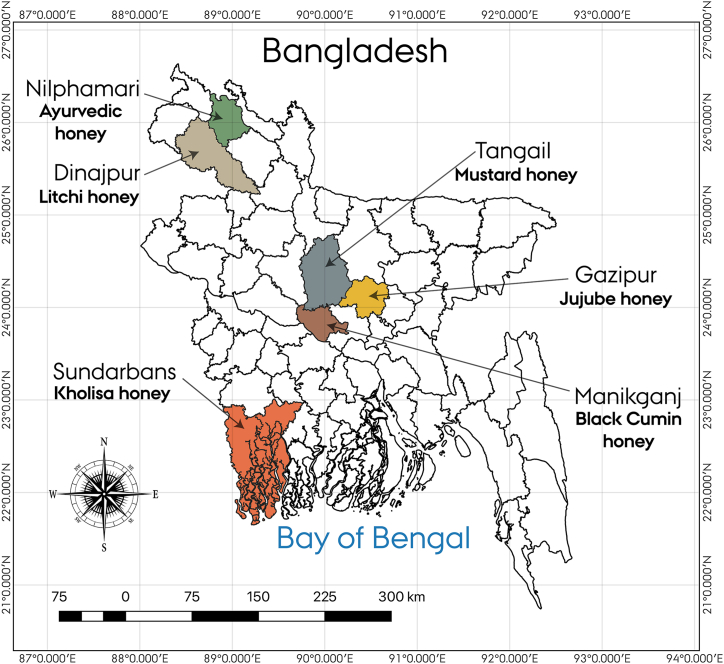
Honey samples collection map. The map was prepared using QGIS software.

**Fig. 2:**
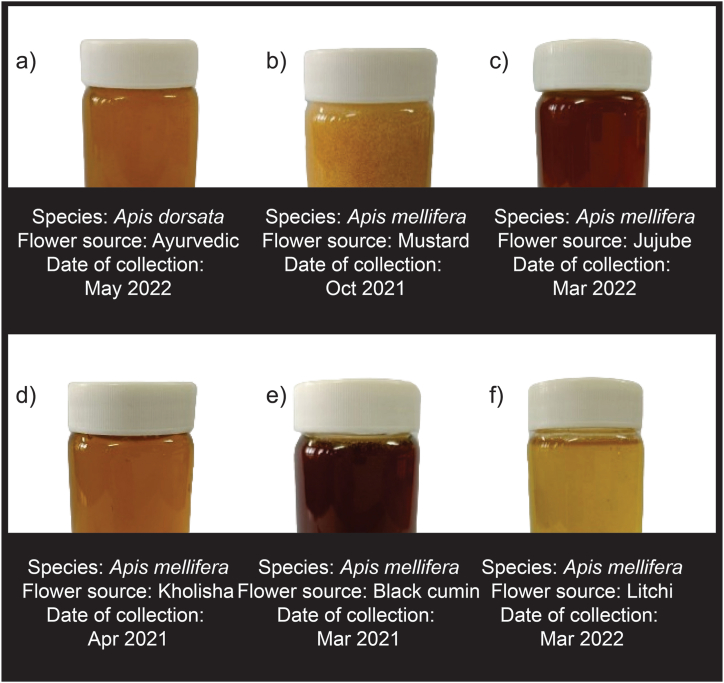
Pictorial view of honey samples collected from different flower sources. a) Ayurvedic honey, b) Mustard honey, c) Jujube honey, d) Kholisha honey, e) Black cumin honey, and f) Litchi honey.

To carry out the experiment, honey samples were collected from a total of six different botanical sources in various regions known for distinct floral sources across Bangladesh (Gazipur, Dinajpur, Nilphamari, Tangail, Manikganj and Sundarbans) between the months of March 2021 and May 2022 as indicated in Fig. 1. Three farms were selected for honey collection from each region, three samples were collected from each farm (Fig. 2). To ascertain the foraging behavior and sources of nectar of *Apis mellifera* and their respective honey, the field was observed for seven days prior to each honey collection. The flower sources were black cumin (*Nigella sativa*), mustard (*Brassica nigra*), litchi (*Litchi chinensis*), jujube (*Ziziphus jujuba* Mill.), kholisha (*Aegiceras corniculatum*) as mono-floral variants while ayurvedic plants was poly-floral variant. These plants were chosen for their representation of mono-floral and poly-floral varieties, reflecting the broad spectrum of Bangladeshi honey and facilitating a comprehensive authentication study across different floral sources.

The honey samples were carefully collected, immediately brough and stored in amber glass containers at 26 °C in the Laboratory of Entomology at Bangabandhu Sheikh Mujibur Rahman Agricultural University. Upon collection, honey samples underwent proper pretreatment procedures to remove any impurities or contaminants. Homogenization and filtration processes were carried out meticulously to maintain sample uniformity.

Samples were stored in amber glass containers at a controlled temperature of 26 °C in the laboratory. Strict adherence to storage conditions minimized the potential for degradation or alteration of sample properties over time.

To analyze the honey's quality parameters, 2000 mL of the raw honey was homogenized, and any resulting foam was subsequently removed. After that, filtration was conducted to eliminate any dead larvae and pupae from the honey samples, and the filtrated honey was also stored in amber glass containers at 26 °C [17].

### 2.2 Honey adulteration

Pure honey was deliberately adulterated by adding sugar syrup at varying concentrations (10 % and 20 % sugar syrup) before undergoing physicochemical analysis [18]. These levels were chosen based on common industry practices and previous studies which indicate that adulteration often occurs within this range to enhance volume and maintain the appearance of natural honey without significantly altering its taste or initial physicochemical properties [13,19,20]. Control sample was marked as A0, while adulterated samples were marked as A10 and A20 indicating 10 % and 20 % sugar syrup adulteration respectively.

Each analysis was performed in triplicate to ensure accuracy and reproducibility of results. Regular calibration of instruments, such as spectrophotometers used for color intensity measurements, pH meters, and conductivity meters, was conducted using standard reference materials to maintain measurement accuracy. Lank samples were included to account for any background contamination or interference in the analyses. Additionally, control samples with known properties were analyzed alongside the honey samples to validate the analytical procedures.

### 2.3 Moisture content

Moisture content of pure and adulterated honey was determined by dividing the difference between dry weight and wet weight by wet weight. The value was then multiplied by 100 and presented as a percentage of moisture content [21]. To ensure consistency, all measurements were conducted under controlled laboratory conditions, with temperature maintained at 26 °C and relative humidity between 50 and 60 %. Samples were homogenized before analysis to ensure uniformity, and measurements were repeated three times for each sample to account for any variability.

### 2.4 Total protein content

Protein content of honey samples was determined using Kjeldahl method [22]. At first honey sample (1 g) was digested using a Kjeldahl digestion flask, which contained 10.0 g of catalyst (a mixture of 9 g of potassium sulphate and 1 g of copper sulphate pentahydrate) and 25 mL of concentrated sulfuric acid for 2.5 h. After digestion, 80 mL of sodium hydroxide was added to each digestion flask. After that, distillation was carried out to trap ammonium hydroxide as ammonium borate in a boric acid solution. Total nitrogen was determined by titration with standardized hydrochloric acid to a mixed indicator endpoint (1 mg mL^−1^ Bromocresol green and 1 mg mL^−1^ methyl red in ethanol).

### 2.5 Ash content

Ash content of honey (5 g) was measured by dividing the whitish gray ash after incinerating the honey samples using an electric heater and muffle furnace at 600 °C for 6 h by the total weight. Ash content was expressed as a percentage by multiplying 100 [23].

### 2.6 Electrical conductivity

Honey's electrical conductivity serves as a valuable indicator for detecting adulteration, whether the honey is derived from nectar (with some variation based on the bee species) or honeydew [24]. Ten grams of honey were added to a 50-mL volumetric flask filled with distilled water for the analysis. The conductivity measurement was recorded once the conductivity stabilized. To prevent contamination between samples, the electrode was rinsed with deionized water and dried with absorbent paper before each new sample was tested.

### 2.7 pH content

Using an analytical balance, the exact weight of 10 g of honey was measured in a 100 mL beaker for the pH analysis. The honey sample was then thoroughly mixed with 75 mL of distilled water. Subsequently, a pH meter (Hanna HI9023; Hanna Instruments, Keysborough, Victoria, Australia) was used to directly measure the pH, with the pH meter calibrated using appropriate buffer solutions (pH 7.0 and pH 4.0) specific to each honey sample, as per the method described by Choudhury, Islam et al. [25].

### 2.8 Color intensity

The color density of the honey was determined following a standardized procedure using a UV spectrophotometer. Readings were taken at two wavelengths, specifically 560 nm and 635 nm, and the honey's color was assessed based on a predefined range. In short, each sample was first diluted with warm deionized water to a concentration of 50 % (w/v). After that, each liter of the resultant solution was passed through a 0.45-μm pore size filter. The absorbance readings were then recorded at 560 nm and 630 nm using a spectrophotometer (Double Beam Spectrophotometer, UH5300, Japan) [24].

### 2.9 Total phenolic content

The Folin–Ciocalteu (FC) method, as explained by Feng, Chitrakar et al. [26], with certain adjustments, was applied to determine the total phenolic content (TPC). 1 g of honey sample was dissolved in 20 mL of deionized water, and 8 mL of this solution was then mixed with 500 mL of FC solution for about 3 min and then 1.5 mL of 25 % sodium carbonate was added to it. The solution was then vortexed and kept at 25 °C for 2 h under dark conditions. Using a spectrometer, the absorbance was measured at 750 nm in relation to the blank (Double Beam Spectrophotometer, UH5300, Japan). To make the calibration curve, Gallic acid (1.25–31.25 μg mL^−1^) was used as the standard, and the TPC was expressed as gallic acid equivalents (GAE) (mg GAE/100 g of honey).

### 2.10 Total flavonoid content

Honey samples (0.5 g) were dissolved in 1 mL of deionized water. The solution was then mixed well with 300 mL of 5 % sodium nitrite. In the above-mentioned solution, another 300 mL of 10 % aluminum chloride was mixed after 5 min and shacked. After about 6 min, the solution was then neutralized with 1M sodium hydroxide 2 mL. To measure total flavonoid content (TFC), using a spectrometer, the absorbance was recoded at 510 nm in against blank sample (Double Beam Spectrophotometer, UH5300, Japan). Catechin (1.25–50.0 μg mL^−1^) was used as the standard, and the TFC was expressed as catechin equivalents (CAE) (mg CAE/100 g of honey) [27].

### 2.11 DPPH antioxidant activity

The antioxidant activity of honey against 1,1-diphenyl-2-picrylhydrazyl (DPPH) radicals was calculated using the method outlined by Rahman, Islam et al. [28] with modifications. After diluting 2 g of honey sample in 10 mL of deionized water, the solution was centrifuged at 3000×*g* and then filtered. Subsequently, 0.1 mL of this solution, at different concentrations (25.0, 50.0, 100, 200, 400, and 800 mg mL^−1^), was mixed with 2.9 mL of methanol (90 μmol L^−1^). The reaction mixture was then allowed to stand for 60 min at room temperature in the dark. The absorbance was measured at 517 nm against a blank using a spectrophotometer (Double Beam Spectrophotometer, UH5300, Japan). The results were expressed as mg ascorbic acid equivalent antioxidant capacity (AEAC) per 100 g of honey (mg AEAC/100 g). This method is well-established for its sensitivity and reliability in measuring the antioxidant capacity of various natural products, including honey.

### 2.12 Hydroxymethylfurfural

Spectrophotometric method was used to measure the amount of hydroxymethylfurfural (HMF) in the standard at a wavelength of 285 nm. To summarize, a 50 mL volumetric flask containing 5 g of honey was dissolved in 25 mL of deionized water. Subsequently, 0.5 mL of Carrez solution I (consisting of 15 g of potassium ferrocyanide dissolved in 100 mL of deionized water) and Carrez solution II (comprising 30 g of zinc acetate dissolved in 100 mL of deionized water) were added and mixed into the solution. After that, deionized water was poured into the flask once more, and a drop of alcohol was added to remove any remaining surface foam. After mixing the solution and filtering it using filter paper, the first 10 mL of filtered liquid were discarded. After filtering, 5 mL of the filtrate (sample) was pipetted into a test tube with 5 mL of deionized water, and 5 mL of the filtrate was combined with 5 mL of 0.2 percent sodium bisulfite solution in a second tube (blank). A vortex mixer was used to completely shake both tubes. Finally, the absorbance of the solutions was measured using a UV–visible spectrophotometer (Double Beam Spectrophotometer, UH5300, Japan) at wavelengths of 284 nm and 336 nm, and this analysis was conducted in quartz cuvettes, in accordance with the method described by Ref. [22]. The HMF content in honey was calculated with equation (1).

(1) $$HMF=\frac{\left(A284-A336\right)\times 74.87}{m}$$

where, A284 = absorbance at 284 nm, A336 = absorbance at 336 nm, 74.87 = calculation factor, m = weight (g).

### 2.13 Statistical analysis for chemometrics

During the experiment data integrity was upheld through stringent record-keeping practices and systematic data analysis. Any outliers or discrepancies in the results were carefully reviewed and verified. The physical and biochemical properties of various mono-floral and poly-floral honeys, including both pure and adulterated samples, were assessed to characterize their components for the purpose of determining authenticity. Data were subjected to analysis of variance (ANOVA) and post hoc analyses were carried out for multiple comparisons. Bonferroni correction was applied to adjust the significance levels during the analysis, which ultimately maintained a stringent criterion for statistical significance across the multiple comparisons conducted. Principal Component Analysis (PCA) was carried out to explore the data with auto-scaled data [29]. Additionally, Pearson correlation was employed to establish the features that were used in determining honey authenticity. All of these statistical analyses were conducted using the “R" statistical software package to ensure robust data interpretation (version 3.1.0, R Foundation, Vienna, Austria) [30].

## 3 Results and discussion

### 3.1 Physico-chemical properties of honey

The choice of 10 % and 20 % sugar syrup adulteration levels is reflective of common practices within the honey industry. Previous research indicates that these levels are frequently used to increase the volume of honey while attempting to retain the sensory and most physicochemical characteristics that appeal to consumers [13,19,20].

Adulteration with 10 % and 20 % sugar syrup is significant enough to impact measurable parameters such as protein content, electrical conductivity, and antioxidant activity, which are critical for authenticity verification. This study's findings shows that adulterated honey displayed distinct deviations in these parameters, further support the notion that common adulteration practices can be effectively detected using the proposed analytical methods.

Significant (p < 0.05) variations were observed for various physicochemical properties including moisture content, pH, optical absorbance, electrical conductivity, protein content, HMF content, total phenolics, flavonoids, and antioxidants in pure and adulterated honey with respect to diverse botanical origins (Table 1, Table 2). Generally, physico-chemical properties of honey samples vary with botanical and geographical origins. Kholisha honey had the highest moisture content (24.07 %), while black cumin honey had the lowest moisture content (17.80 %). With the adulteration, the moisture content increased significantly, regardless of botanical variations. Bangladeshi honey generally exhibited a moisture content range consistent with international standards for honey quality. However, variations exist due to local climatic conditions affecting floral availability and nectar concentration. The botanical origin, the time of year honey is harvested, the level of hive maturity attained, the processing methods used, and the storage environment (e.g. temperature, light, O_2_, etc.) are some of the variables that affect honey's water content [31]. The moisture content in honey investigated by Khalil, Moniruzzaman et al. [32] was between 11.59 and 14.13 %.

**Table 1:** Moisture, protein, ash content and electrical conductivity of honey samples.

Flower sources	^∗^Adulteration	Moisture (%)	Protein (%)	Ash (%)	Electrical conductivity (mS/cm)
Ayurvedic	A0	20.76 ± 0.25^c^	9.16 ± 0.02^a^	0.53 ± 0.03^a^	0.41 ± 0.005^a^
	A10	23.67 ± 0.15^b^	8.80 ± 0.07^b^	0.25 ± 0.19^ab^	0.38 ± 0.00^b^
	A20	25.80 ± 0.34^a^	7.94 ± 0.03^c^	0.10 ± 0.15^b^	0.42 ± 0.00^a^
Black Cumin	A0	17.80 ± 0.26^c^	11.53 ± 0.28^a^	0.08 ± 0.02^a^	1.53 ± 0.02^a^
	A10	20.02 ± 0.07^b^	9.03 ± 0.04^b^	0.08 ± 0.004^a^	1.46 ± 0.06^a^
	A20	24.99 ± 1.01^a^	8.07 ± 0.80^b^	0.03 ± 0.03^a^	1.39 ± 0.15^a^
Jujube	A0	21.08 ± 0.24^c^	11.30 ± 0.01^a^	0.92 ± 0.20^a^	1.27 ± 0.00^a^
	A10	25.13 ± 0.41^b^	9.05 ± 0.04^b^	0.79 ± 0.162^a^	1.24 ± 0.13^a^
	A20	26.23 ± 0.05^a^	7.07 ± 0.06^c^	0.69 ± 0.08^a^	1.20 ± 0.00^a^
Kholisha	A0	24.07 ± 0.13^c^	1.32 ± 0.47^a^	0.44 ± 0.31^a^	0.49 ± 0.005^a^
	A10	25.94 ± 0.19^b^	1.01 ± 0.010^a^	0.041 ± 0.02^a^	0.45 ± 0.00^a^
	A20	27.46 ± 0.57^a^	0.03 ± 0.004^b^	0.05 ± 0.002^a^	0.45 ± 0.00^a^
Litchi	A0	21.37 ± 0.24^c^	3.80 ± 0.24^a^	0.61 ± 0.35^a^	0.24 ± 0.01^a^
	A10	25.95 ± 0.15^b^	0.15 ± 0.23^b^	0.36 ± 0.10^a^	0.22 ± 0.00^b^
	A20	27.13 ± 0.28^a^	0.09 ± 0.00^b^	0.18 ± 0.11^a^	0.25 ± 0.005^a^
Mustard	A0	20.95 ± 0.05^c^	1.14 ± 0.03^a^	0.63 ± 0.13^a^	0.30 ± 0.01^a^
	A10	22.80 ± 0.26^b^	0.95 ± 0.05^b^	0.41 ± 0.02^b^	0.26 ± 0.00^b^
	A20	26.16 ± 0.98^a^	0.033 ± 0.04^c^	0.13 ± 0.01^c^	0.29 ± 0.00^a^

^∗^A0, A10 and A20 represents control, 10 % and 20 % adulteration of honey respectfully. Data presented as mean ± standard deviation. Different superscript letters in the same column within each group represents significant differences (p < 0.05).

**Table 2:** Total phenol, total flavonoid, antioxidants and hydroxymethylfurfural content of honey samples.

Flower sources	^∗^Adulteration	TPC (mg GAE/100 g)	TFC (mg CAE/100 g)	Antioxidant (mg AEAC/100g)	Hydroxymethylfurfural (mg/kg)
Ayurvedic	A0	53.47 ± 0.60^a^	5.75 ± 0.03^a^	7.30 ± 0.10^a^	227.71 ± 0.05^a^
	A10	53.41 ± 0.33^a^	5.30 ± 0.29^b^	6.77 ± 0.34^ab^	167.93 ± 0.00^b^
	A20	51.90 ± 0.12^b^	5.23 ± 0.005^b^	6.28 ± 0.11^b^	116.35 ± 2.92^c^
Black Cumin	A0	64.05 ± 0.89^a^	7.90 ± 0.01^a^	4.82 ± 0.005^a^	227 ± 0.04^a^
	A10	63.70 ± 0.34^a^	7.62 ± 0.10^b^	3.77 ± 0.17^b^	223.52 ± 8.47^a^
	A20	61.28 ± 0.33^b^	7.45 ± 0.03^c^	3.49 ± 0.20^b^	221.01 ± 8.55^a^
Jujube	A0	147.85 ± 0.94^a^	17.00 ± 0.45^a^	3.42 ± 0.01^a^	123.96 ± 7.04^a^
	A10	146.27 ± 0.91^a^	16.67 ± 0.37^a^	3.22 ± 0.07^ab^	117.80 ± 3.63^a^
	A20	133.50 ± 16.90^a^	16.44 ± 0.05^a^	3.20 ± 0.12^b^	117.78 ± 9.77^a^
Kholisha	A0	45.83 ± 0.14^a^	9.63 ± 0.39^a^	5.96 ± 0.005^a^	216.85 ± 0.77^a^
	A10	44.46 ± 0.56^b^	9.49 ± 0.32^a^	5.07 ± 0.06^b^	181.92 ± 17.38^a^
	A20	41.76 ± 0.11^c^	9.43 ± 0.41^a^	4.53 ± 0.47^b^	146.69 ± 70.25^a^
Litchi	A0	56.17 ± 0.12^a^	6.69 ± 0.27^a^	9.23 ± 0.35^a^	142.62 ± 9.21^a^
	A10	54.46 ± 0.14^b^	6.68 ± 0.45^a^	8.27 ± 0.02^b^	121.78 ± 0.00^a^
	A20	54.18 ± 0.87^b^	6.66 ± 0.40^a^	8.07 ± 0.02^b^	116.52 ± 2.46^a^
Mustard	A0	33.18 ± 0.26^a^	4.39 ± 0.03^a^	4.50 ± 0.13^a^	231.86 ± 6.49^a^
	A10	32.13 ± 0.25^b^	4.36 ± 0.03^a^	3.42 ± 0.71^a^	130.29 ± 9.10^b^
	A20	31.46 ± 0.35^b^	4.20 ± 0.13^a^	3.33 ± 0.50^a^	97.87 ± 6.27^c^

^∗^A0, A10 and A20 represents control, 10 % and 20 % adulteration of honey respectfully. Data presented as mean ± standard deviation. Different superscript letters in the same column within each group represents significant differences (p < 0.05).

Pure black cumin honey had the highest protein content of 11.53 %, followed by jujube honey (11.30 %), while kholisha and mustard honey exhibited very low protein levels (1.32 % and 1.14 % respectively). The protein content of adulterated honey samples showed variations based on both the level of adulteration and the botanical source. Among the adulterated honey samples, 10 % and 20 % adulterated litchi, kholisha, mustard, black cumin, jujube, and ayurvedic honey showed significantly less protein content than pure honey (Table 1). The protein content in our tested samples, particularly in specific floral types like black cumin and jujube honey, was generally higher compared to some international honeys. This might be attributed to the protein profiles of the dominant floral sources prevalent in Bangladesh.

Ash content is indicative of the mineral content in honey and can vary based on botanical sources and adulteration levels. Pure jujube honey retained the maximum ash content of 0.92 %, followed by mustard honey (0.63 %), whereas black cumin honey had the minimum ash content (less than 0.1 %). 10 % and 20 % adulteration with sugar syrup overall reduced the ash content, however, the reduction was not significant in black cumin, jujube, kholisha, and litchi honey. A significant difference was observed in adulterated mustard honey samples (Table 1).

EC is a critical parameter in determining the botanical origin of honey since it is closely related to the concentration of mineral salts, organic acids, and proteins [33]. Different types of honey exhibit specific ranges of EC values, which can serve as indicators of their floral sources. For instance, honey derived from nectar generally shows lower EC values compared to honeydew honey, which is rich in minerals and thus has higher EC [34]. Pure honey typically has the following EC thresholds: Acacia honey-often exhibits low EC values, around 0.08–0.30 mS/cm, Forest honey - often exhibits EC values around 0.09–1.99 mS/cm, Poly-floral honeys - these can have more variable EC values but usually fall within 0.09–0.74 mS/cm depending on the predominant floral sources [35]. Electrical conductivity levels showed notable differences when compared to honeys from countries such as Italy or New Zealand, primarily due to the varied mineral content influenced by regional flora and soil types.

In the present study, black cumin honey demonstrated the highest EC of 1.53 mS/cm, followed by jujube honey (1.27 mS/cm), indicating a rich mineral content similar to that of honeydew honeys. Conversely, litchi honey exhibited the lowest EC value at 0.24 mS/cm, characteristic of nectar honeys. The addition of sugar syrup did not significantly affect the electrical conductivity except for ayurvedic, litchi, and mustard honey (Table 1). The EC was 0.74 ± 0.07 mS/cm and the ash content was 0.32 ± 0.05 percent (significantly different, p < 0.05) when compared to the results obtained by Oddo, Piazza et al. [36] for strawberry tree honey from Italy.

The significance of the threshold values lies in their ability to authenticate honey based on its floral source. For instance, an unexpectedly high EC value in a honey labeled as Acacia suggests possible adulteration or mislabeling. By comparing the EC values of our samples to these known thresholds, we provide a robust method for verifying the botanical origin of honey.

The highest phenolic compound content was also found in jujube honey (147.85 mg GAE/100g), while mustard honey had the lowest phenolic compound content (33.18 mg GAE/100g). Adulteration with sugar syrup significantly reduced total phenolic content in all samples except for jujube honey (Table 2). Similar to phenolic content, among the pure honey samples tested, jujube honey had the highest flavonoid content (17 mg CAE/100g), while mustard honey had the lowest content of flavonoid (4.39 mg CAE/100g). A decreasing trend in flavonoid content was observed with the increase in adulteration percentage. Total flavonoid significantly decreased in ayurvedic and black cumin honey (Table 2).

Other studies, such as the one conducted by Al, Daniel et al. [37] on Romanian mono-floral honey and honeydew, reported phenolic values ranging from 2 to 125 mg GAE/100g of honey. Additionally, research by Meda, Lamien et al. [38] indicated that floral honeys and multifloral honeys exhibited a broader range of total phenolic content, spanning from 32.59 to 114.75 mg GAE/100g of honey. For the flavonoid content of pure honey, statistically the highest value was observed in black cumin honey, which is 1.4 mg CAE/100g followed by jujube honey, which is 0.3 mg CAE/100g. Similar results have been obtained by Meda, Lamien et al. [38] and Al, Daniel et al. [37] for various varieties of honey. The range they have found in their research is between 0.17 and 8.35 mg CAE/100g of honey (for example, poly-floral) and between 0.91 and 15.33 mg CAE/100g of honey (for example, mono-floral).

The highest antioxidant levels were found in litchi honey, exhibiting significantly higher antioxidant activity (9.23 mg AEAC/100 g) compared to other honey samples. Jujube honey possessed the lowest antioxidant activity of 3.42 mg of AEAC/100g. The DPPH assay, which measures the radical scavenging activity of antioxidants, was used for this analysis. This method is recognized for its robustness and accuracy in evaluating the antioxidant potential of food substances. It is particularly reliable in distinguishing between pure and adulterated honey because it quantifies the capacity of honey to neutralize free radicals, a property that is often compromised in adulterated samples [39]. The antioxidant levels and phenolic content in Bangladeshi honey were observed to be relatively high, especially in litchi honey. This suggests a rich presence of bioactive compounds, possibly reflective of the country's unique floral diversity.

Adulterated honey typically exhibits lower antioxidant activity due to the dilution of bioactive compounds that contribute to this property. In present study, significant decreases in antioxidant levels were observed with increasing adulteration, supporting the reliability of the DPPH method in detecting purity. For example, litchi honey showed a marked reduction in antioxidant activity with both 10 % and 20 % adulteration, thereby confirming the sensitivity of this method in identifying adulterated samples.

Regarding the antioxidant potential, litchi honey displayed significantly higher antioxidant activity of 9.23 mg of the ascorbic acid equivalent antioxidant capacity AEAC/100g over the other sources, With the adulteration percentage, antioxidant activity decreased significantly in most of the honey samples except mustard honey (Table 2). The majority of the substances in honey that have an antioxidant property are phenolic compounds [[40], [41], [42]]. Results from Silici, Sagdic et al. [43]'s analysis of samples of Turkish Rhododendran honey's antioxidant capacity ranged from 12.76 to 80.80 mg of AEAC/100 g of honey, exceeding our study's findings.

Monitoring HMF levels in honey is essential for verifying product quality, meeting regulatory expectations, and safeguarding consumer health. The highest HMF content was observed in pure ayurvedic and black cumin honey (227.71 mg/kg), followed by mustard and kholisha honey (231.86 and 216.85 mg/kg), whereas the least HMF content was recorded in jujube honey (123.96 mg/kg) (Table 2). Similar to phenolic and flavonoid content, a decreasing trend was observed in HMF value with the addition of sugar syrup in all honey samples.

Exceeding EU limits for HMF content may raise compliance issues with regulatory standards and quality requirements.

The findings regarding the HMF content in the analyzed honey samples were alarming, as they exceeded the limits set by the European Union in 2001. Among the adulterated honey samples, 20 % adulterated ayurvedic honey had the highest HMF content, exceeding 200, while the lowest HMF content among adulterated honeys was found in 20 % adulterated mustard honey, which exceeded 140. In pure honeys, black cumin honey had the highest HMF content, exceeding 150, followed by kholisha and ayurvedic honeys, both with HMF levels below 150. These results were consistent with the findings of other researchers, such as Turhan [44] and Khalil, Sulaiman et al. [45], who observed a progressive increase in HMF levels in honey stored for 8–12 months. This increase in HMF content was attributed to changes in the pH and acidity of honey. Studies have also indicated that HMF may have genotoxic effects and mutagenic potential, as highlighted by Khalil, Sulaiman et al. [45] and Capuano and Fogliano [46].

Elevated HMF levels can impact the sensory attributes of honey, leading to changes in color, flavor, and aroma. The formation of HMF is associated with the caramelization of sugars, resulting in a darker color and altered taste profile. Honey with high HMF levels may be perceived as lower quality due to these sensory changes, affecting consumer perception and acceptance. Beyond quality aspects, elevated HMF levels in honey can raise safety concerns. HMF is classified as a byproduct of sugar degradation and has been linked to potential health risks. Studies suggest that HMF may have genotoxic and carcinogenic properties, prompting regulatory authorities like the EU to establish maximum limits to safeguard consumer health. The presence of high HMF levels in honey signals the importance of stringent quality control measures and appropriate storage practices to prevent excessive heat exposure or prolonged storage periods. Adhering to recommended storage conditions, maintaining proper temperature control, and monitoring processing methods are crucial to minimize the formation of HMF and ensure honey quality and safety.

### 3.2 Differences in pH of honey

**Fig. 3:**
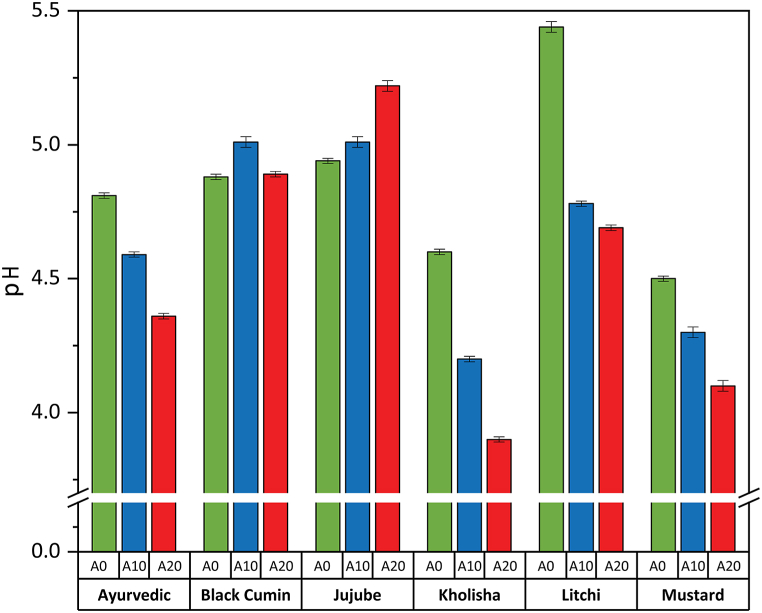
pH of different honey samples. A0, A10 and A20 represents control, 10 % and 20 % adulteration of honey respectfully.

The pH value of honey can provide insights into its acidity or alkalinity [47]. Pure litchi honey had the highest pH level (5.44), followed by jujube (4.94) and black cumin honey (4.88) (Fig. 3). With the increase in adulteration percentage, the pH value increased in black cumin and jujube, while a decreasing trend was found in ayurvedic, kholisha, litchi, and mustard honey. Jiménez, Mateo et al. [48] also noticed a non-significant increase in pH in honey samples after two years of room temperature storage, and they reported similar results.

This value falls within the permissible range for nectar honey (from 3.2 to 4.5); results showed a significant difference (p < 0.05) achieved by Oddo, Piazza et al. [36] for strawberry tree honey (from 4.0 to 4.4, a mean 4.2 ± 0.1). For the most part, honey is naturally acidic, regardless of where it comes from. This parameter affects texture, stability, and storage, making it crucial during extraction and storage [33,49].

### 3.3 Optical absorbance of honey samples

**Fig. 4:**
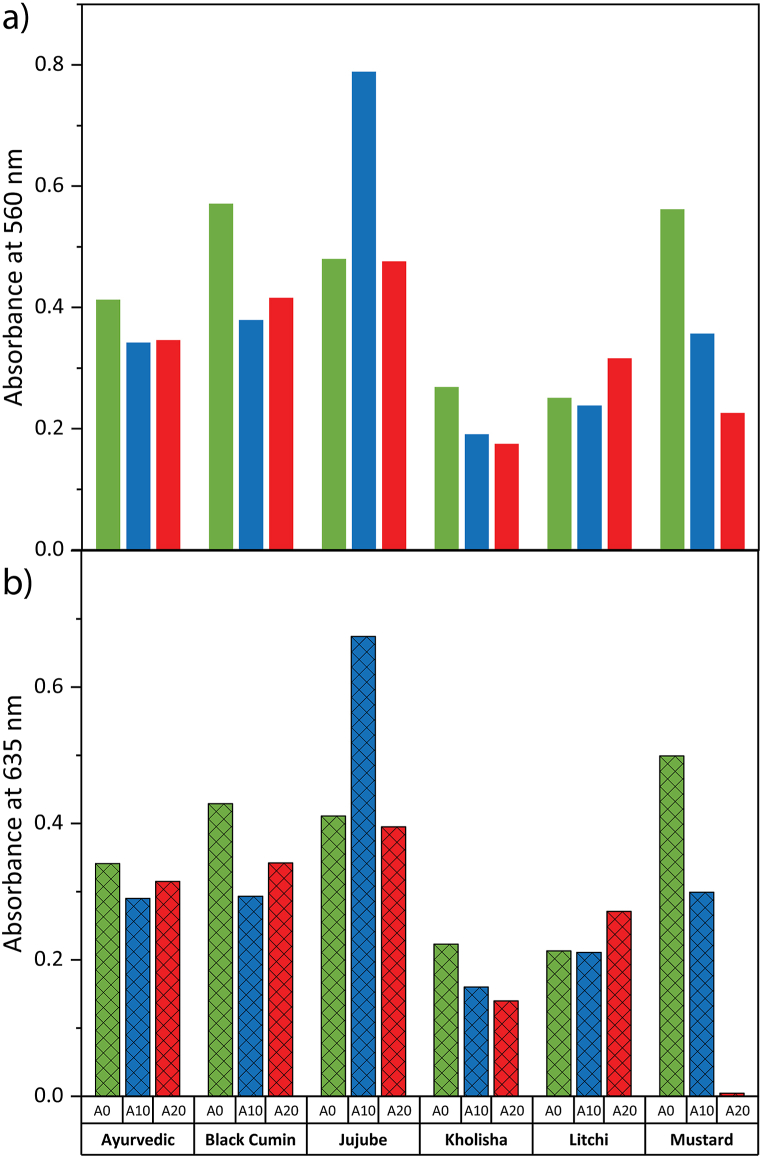
Optical absorbance at a) 560 nm, and b) 635 nm.

Moreover, jujube pure honey exhibited the highest color intensity at both 560 nm and 635 nm, followed by black cumin and mustard honey (Fig. 4). The color intensity of honey at specific wavelengths (560 nm and 635 nm) provides information about its visual characteristics [50,51]. Among the adulterated honey samples, 10 % adulterated jujube honey exhibited the highest color intensity at both wavelengths, which confirms its amber color, while 20 % adulterated kholisha honey displayed the lowest at 560 nm, meaning it is the darkest among the others. Among pure honey, litchi honey showed moderate light color intensity (Fig. 4). These results reflect variations in honey color due to botanical sources and adulteration levels. In summary, the study demonstrates that the physicochemical and biochemical properties of adulterated honey samples can vary significantly based on the botanical source and extent of adulteration. These findings emphasize the importance of comprehensive analysis and authentication to ensure honey quality and authenticity on the market.

As honey ages, its color may also change due to beekeeping interventions and other conservation techniques including using ancient honeycombs, coming into contact with metals, or being exposed to extreme heat or light. Natural variations in honey's color occur in a broad spectrum of tones, from light yellow to amber, dark amber and black, and in rare instances, even red or green tints [52]. The color intensity at 560 nm of jujube pure honey was found statistically highest (0.6) means its color is Amber followed by pure honey of black cumin (0.5) and mustard (0.35) means light amber in color. Also, the color intensity at 635 nm of jujube pure honey (0.5) was found statistically highest, which means amber in color followed by pure honey of black cumin (0.35) and mustard (0.29) means light amber in color*.* In color intensity at 560 nm and 635 nm, 10 % adulterated jujube honey (0.7) statistically highest among other samples, and 20 % adulterated kholisha honey is the lowest (0.1). The color variation observed in fresh honeys was ascribed to either the oxidation of polyphenols or an increase in melanoidin chemicals (the Maillard process), which give honey a brown hue and cause significant alterations overall (quinines) [48,53]. Certain Italian, Slovenian, and Indian honeys were reported to have ABS_450_ values of 25–3413 mAU, 70–495 mAU, and 524–1678 mAU, respectively [54], which are more or less similar with our study. Their mineral content is one of the key variables that determines their color characteristics. Dark honeys often include more ash than light honeys, which typically have lower ash concentrations [55]. In the results, pure Kholisha honey is lighter than others and it has high ash content than pure black cumin honey.

### 3.4 Heatmap analysis

**Fig. 5:**
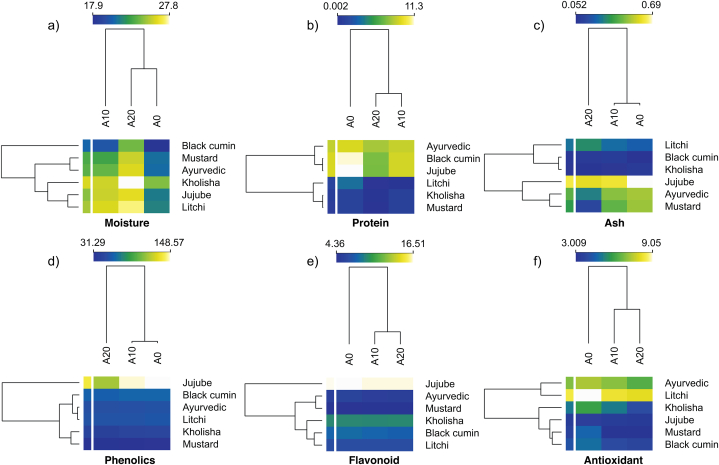
Heatmap resuming the effects of adulteration on a) moisture, b) protein, c) ash, d) total phenolics, e) total flavonoids content, and f) antioxidants capacity.

The variation in moisture, protein, ash, total phenolics, total flavonoids, and antioxidants among the pure and adulterated honey samples is summarized in the heatmaps illustrated in Fig. 5. Each cell in the heatmap is color-coded to represent the magnitude of a specific physicochemical or biochemical parameter for different honey samples. In Fig. 5, lighter colors (e.g., white) indicate higher values, while darker colors (e.g., blue) suggest lower values. The rows and columns of the heatmap represent the honey samples and the level of adulteration, respectively. Both rows and columns were organized using hierarchical clustering, which grouped similar items based on types and adulteration. This clustering helps to visually highlight patterns, such as which samples have similar profiles or which adulteration tend to be associated together.

The heatmap allowed for quick visual identification of patterns or trends across samples. For instance, a cluster of light-colored cells indicated a group of honey samples with high antioxidant activity. The clustering according to the adulteration proved that the higher percentage of adulteration increased moisture content while decreasing other properties tested (Fig. 5). The analysis of moisture, protein, ash, phenolics, flavonoids, and antioxidant content results showed that these parameters varied between 18 and 28 %, 0.002–11 %, 0.05–0.69 %, 31–149 GAE/100g, 4–17 CAE/100g, and 3–9 AEAC/100g, respectively. Clustering according to the types of honey showed that the highest moisture was determined for those adulterated with 20 % sugar syrup. From the heat map and associated dendrogram, it can be easily found that there are similar samples in both adulteration and flower source categories based on specific parameters. Jujube honey turned out to be a single group in terms of phenolics and flavonoids. For phenolics, A20 turned out to be different from A0 and A10, while, for flavonoids and antioxidants, A0 turned out to be different from the adulterated one. In terms of protein content, ayurvedic, black cumin, and jujube were similar and had the highest protein contents. By understanding these patterns and relationships in the heatmap, we deduced information about the similarities and differences between honey samples from various floral sources, the impact of adulteration, or the unique biochemical signatures of regional honeys.

### 3.5 Principal component analysis

**Fig. 6:**
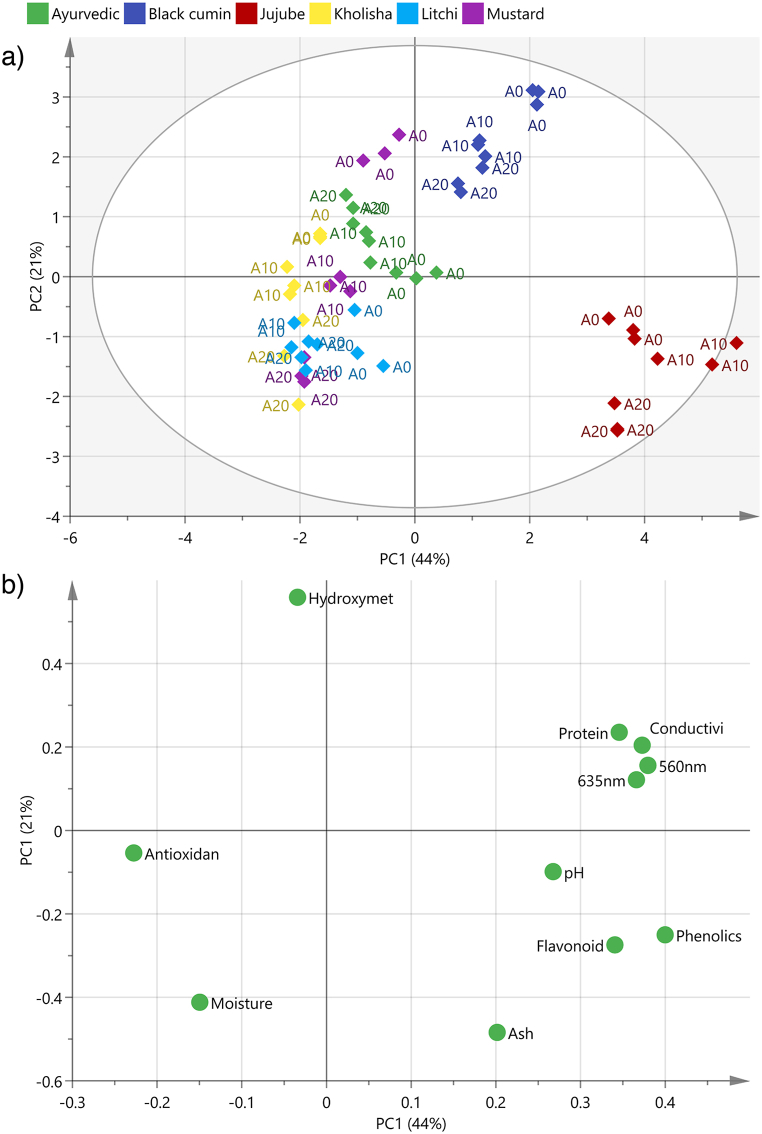
Principal component analysis representing relations of honey parameters from different nectarine honey sources. a) score plot, b) loading plot.

A principal component analysis (PCA) was performed to investigate data variation [56]. According to PCA analysis, principal component (PC) 1 contributed 44 % and PC 2 contributed 21 % of the variation, meaning that the first two components could explain more than 65 % of the variation (Fig. 6). The number of components were chosen using the scree plot (not shown).

Honey from different floral sources were marked using distinguishable colors and the level of adulteration was marked on each sample in score plot (Fig. 6a). It can be seen that honey samples from different flower origins made separate clusters in the score plot. In a score plot, samples that are situated closely are similar in nature. On the PCA space, the adulterated samples from different flower sources stayed together in different clusters. The influence of variables on PCA is represented in loading plot Fig. 6b. Parameters that are lies together are correlated in loading plot. Protein content, electrical conductivity, and absorbance at 560 nm and 635 nm positively contributed in both PC 1 and PC 2. Conversely, antioxidant and moisture content negatively contributed to both dimensions. Ash content, pH value, total phenolic content, and total flavonoid content were found to be positively correlated. Similarly, protein content, electrical conductivity, and color intensities at 560 nm and 635 nm were found to be positively correlated. Moisture content and antioxidant activity were negatively correlated with all the other variables, while HMF was negatively correlated with various bioactive compounds.

Jujube honey was mostly attributed to phenolics and flavonoids, while black cumin honey was mostly attributed to higher protein contents. Ayurvedic, litchi, and kholisha honey were mostly attributed to higher antioxidants. Mustard and black cumin honey were attributed to lower ash contents. Advanced chemometric tools has been recently used by Cherigui, Chikhi et al. [57] to authenticate honey purity. The findings in this study provide insights into the key factors driving variation in honey characteristics from different floral sources, and will be helpful in developing advanced models to authenticate honey purity.

### 3.6 Correlation analysis

**Fig. 7:**
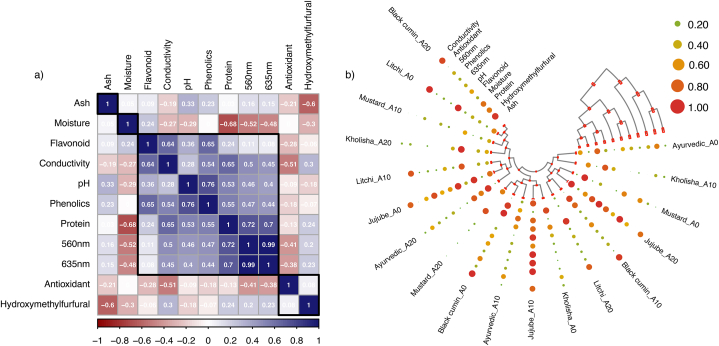
Correlation and cluster analysis plot. a) correlation analysis, b) cluster analysis.

Pearson correlation analysis shows the statistical clustering of pure and adulterated honey with their biochemical and physicochemical parameters (Fig. 7). Understanding these correlations can provide valuable insights into the interdependencies of honey characteristics, contributing to a more comprehensive assessment of honey quality and authenticity [58]. Each cell in the correlation matrix represents the correlation coefficient between two parameters, ranging from −1 (perfect negative correlation) to +1 (perfect positive correlation). Positive values indicate that as one parameter increases, the other tends to increase, while negative values suggest an inverse relationship.

From Fig. 7a, it can be seen that moisture content, ash content, antioxidants and HMF do not have very high positive correlations with other variables. Mainly, moisture content and antioxidants have a negative correlation with other variables tested, while ash contents and HMF do have a very low positive correlation. Parameters such as total phenolic content and antioxidant activity showed strong positive correlations, indicating that they often increase or decrease together. Similar strong correlations were found between electrical conductivity and protein content. These correlations provide insights into potential shared underlying mechanisms or influences affecting honey characteristics, such as floral sources impacting both phenolic content and antioxidants similarly. Fig. 7b presents the cluster matrix of honey samples based on correlation coefficients. Similar pattern can be observed as Fig. 7a. A similar correlation between antioxidants, polyphenols and flavonoids was found in honey from Tunisia [59], Europe [60], Turkey [61] and Brazil [62].

While some results, such as the increase in moisture content with adulteration, aligned with our expectations and existing literature, there were indeed unexpected findings that warrant further investigation. One of the unexpected findings was the level of variability in antioxidant activity among the different honey samples, especially in adulterated samples. For instance, despite increased adulteration usually leading to a reduction in antioxidant levels, some samples maintained relatively stable antioxidant activities. This anomaly could be attributed to specific floral sources or other bioactive components not fully accounted for in our current analysis. Another surprising result was the significant fluctuation in protein content across various floral sources upon adulteration. While a decrease was anticipated, the rates of changing were inconsistent, suggesting possible interactions between adulterants and certain floral compounds that required deeper exploration. The elevated HMF levels in certain honey samples, exceeding European Union limits, were also unexpected. The correlation between storage conditions, pH changes, and HMF formation suggests that more research is needed into the underlying chemical processes and conditions contributing to these high levels.

## 4 Conclusion

The authentication of honey is essential for advancing the apiculture sector and ensuring the quality and safety of honey products in the market. This study demonstrated that by profiling physicochemical and bioactive properties, such as moisture, pH, protein, ash content, and antioxidant activity, a comprehensive assessment of honey's authenticity and purity can be achieved. The analysis of these parameters provided crucial insights into distinguishing between pure and adulterated honeys and identifying their botanical origins. This research established a foundational system for honey authentication in Bangladesh, using methods that are both accessible and effective for local producers and regulatory bodies.

While the botanical origins typically determine honey's nutritional value, this study emphasizes the importance of additional biochemical indicators in the authentication process. Moreover, employing multivariate analysis techniques helped identify patterns and correlations among various honey samples, enhancing our understanding of honey composition and its authenticity.

Future research should aim to incorporate rapid authentication systems, such as optical and biosensors or spectroscopic techniques, to further refine and expedite the process. By addressing these aspects, the potential for broader implementation of honey authentication methods can be increased, ultimately improving consumer confidence and expanding export opportunities for Bangladeshi honey.

## CRediT authorship contribution statement

**Roksana Al Nafiu Insha:** Writing – review & editing, Writing – original draft, Methodology, Investigation, Formal analysis. **Md Nahidul Islam:** Writing – review & editing, Writing – original draft, Visualization, Validation, Supervision, Methodology, Formal analysis, Conceptualization. **Joydeb Gomasta:** Writing – review & editing, Validation, Supervision, Investigation. **Mohammad Nazmol Hasan:** Validation, Investigation, Data curation. **Md Ruhul Amin:** Validation, Supervision, Resources, Investigation. **Noor Shaila Sarmin:** Writing – review & editing, Supervision, Resources, Methodology, Investigation, Data curation. **Md Mamunur Rahman:** Writing – review & editing, Writing – original draft, Supervision, Project administration, Methodology, Funding acquisition, Conceptualization.

## Consent to participate

Not Applicable.

## Availability of data and material

The data that support the findings of this study are available on request from the corresponding author. The data are not publicly available due to privacy or ethical restrictions.

## Ethics approval

Not Applicable.

## Consent for publication

Not Applicable.

## Code availability

Not Applicable.

## Declaration of competing interest

The authors declare that they have no known competing financial interests or personal relationships that could have appeared to influence the work reported in this paper.
